# Regulation of Noise-Induced Loss of Serotonin Transporters with Resveratrol in a Rat Model Using 4-[^18^F]-ADAM/Small-Animal Positron Emission Tomography

**DOI:** 10.3390/molecules24071344

**Published:** 2019-04-05

**Authors:** I-Hsun Li, Jui-Hu Shih, Yun-Tin Jhao, Hsin-Chien Chen, Chuang-Hsin Chiu, Chien-Fu F. Chen, Yuahn-Sieh Huang, Chyng-Yann Shiue, Kuo-Hsing Ma

**Affiliations:** 1 Department of Pharmacy Practice, Tri-Service General Hospital, Taipei 114, Taiwan; lhs01077@gmail.com (I.-H.L.); jtlovehl@gmail.com (J.-H.S.); 2 School of Pharmacy, National Defense Medical Center, Taipei 114, Taiwan; 3 Department of Biology and Anatomy, National Defense Medical Center, Taipei 114, Taiwan; ytcola581470@gmail.com (Y.-T.J.); anatoman2001@yahoo.com.tw (Y.-S.H.); 4 Department of Otorhinolaryngology-Head and Neck Surgery, Tri-Service General Hospital, National Defense Medical Center, Taipei 114, Taiwan; acolufreia@yahoo.com.tw; 5 Department of Nuclear Medicine, Tri-Service General Hospital, National Defense Medical Center, Taipei 114, Taiwan; treasure316@gmail.com (C.-H.C.); shiue@ntuh.gov.tw (C.-Y.S.); 6 Graduate Institute of Life Sciences, National Defense Medical Center, Taipei 114, Taiwan; t70cyy@yahoo.com

**Keywords:** serotonin transporters, resveratrol, noise-induced hearing loss, 4-[^18^F]-ADAM, small-animal PET

## Abstract

Serotonin (5-HT) plays a crucial role in modulating the afferent fiber discharge rate in the inferior colliculus, auditory cortex, and other nuclei of the ascending auditory system. Resveratrol, a natural polyphenol phytoalexin, can inhibit serotonin transporters (SERT) to increase synaptic 5-HT levels. In this study, we investigated the effects of resveratrol on noise-induced damage in the serotonergic system. Male Sprague-Dawley rats were anaesthetized and exposed to an 8-kHz tone at 116 dB for 3.5 h. Resveratrol (30 mg/kg, intraperitoneal injection [IP]) and citalopram (20 mg/kg, IP), a specific SERT inhibitor used as a positive control, were administered once a day for four consecutive days, with the first treatment occurring 2 days before noise exposure. Auditory brainstem response testing and positron emission tomography (PET) with *N*,*N*-dimethyl-2-(2-amino-4-[^18^F]fluorophenylthio)benzylamine (4-[^18^F]-ADAM, a specific radioligand for SERT) were used to evaluate functionality of the auditory system and integrity of the serotonergic system, respectively, before and after noise exposure. Finally, immunohistochemistry was performed 1 day after the last PET scan. Our results indicate that noise-induced serotonergic fiber loss occurred in multiple brain regions including the midbrain, thalamus, hypothalamus, striatum, auditory cortex, and frontal cortex. This noise-induced damage to the serotonergic system was ameliorated in response to treatment with resveratrol and citalopram. However, noise exposure increased the hearing threshold in the rats regardless of drug treatment status. We conclude that resveratrol has protective effects against noise-induced loss of SERT.

## 1. Introduction

Noise-induced hearing loss is one of the most prevalent occupational health hazards worldwide [1]. Exposure to intense, loud noise contributes to afferent dendrite swelling underneath the inner hair cells and mechanical damage to the outer hair cells [2]. Glutamate, the major excitatory neurotransmitter for inner hair cell–auditory nerve synapses, has been reported to cause noise-induced dendrite damage [3]. Glutamate excitotoxicity has been suggested as being attributed to noise-induced hearing loss. Moreover, various mechanisms underlying noise-induced adjacent neuronal death caused by glutamate excitotoxicity have been proposed, including elevation of intracellular calcium, accumulation of oxidizing free radicals, impairment of mitochondrial function, and activation of apoptotic and autophagic programs [4]. In our previous study, we found that serotonergic fibers were markedly reduced by 30–52% in various rat brain regions 4 weeks after noise exposure (8-kHz noise at 118 dB of sound pressure level [SPL] for 3.5 h), suggesting that noise-induced hearing loss involves a reduction in serotonin transporter (SERT) expression [5]. 

Serotonergic fibers originating in raphe nuclei are known to project into several auditory structures, including the cochlear nucleus, auditory cortex, and inferior colliculus [6,7,8,9,10,11,12,13,14]. Glutamate transmission has been reported to be indirectly modulated by the serotonin (5-HT) system through 5-HT_1A_, 5-HT_1B_, 5-HT_3_, and 5-HT_7_ receptors as well as SERT [15]. Furthermore, the use of citalopram, a selective inhibitor of SERT, has demonstrated a positive effect on auditory processes in elderly patients who previously exhibited low performance in auditory processes [16]. However, the protective role of SERT inhibitors against noise-induced hearing loss and serotonergic neuronal damage remains unclear.

Resveratrol is a natural polyphenolic phytoalexin that widely exists in plant species, such as grapes, peanuts, and mulberries [17]. Resveratrol possesses several pharmacological properties, including anti-inflammation [18], antioxidation [19], and neuroprotection [20]. Resveratrol inhibited the uptake of [^3^H]-5-HT by synaptosomes in the rat brain [21] and displayed antidepressant-like activity in a chronic stress model in rodents through selective inhibition of MAO-A compared with MAO-B to regulate 5-HT and noradrenaline levels [22,23]. In addition, a positron emission tomography (PET) imaging study demonstrated that resveratrol had partial SERT binding potential in vivo using *N*,*N*-dimethyl-2-(2-amino-4-[^18^F] fluorophenylthio)benzylamine (4-[^18^F]-ADAM, a specific radioligand for SERT) [24]. Therefore, in this study, we investigated the protective effects of resveratrol and citalopram, a positive control, through pharmacological manipulation of the central 5-HT system against noise-induced loss of serotonergic fibers in an animal model.

## 2. Results

### 2.1. High Intensity Noise Induced Permanent Hearing Loss

**Figure 1:**
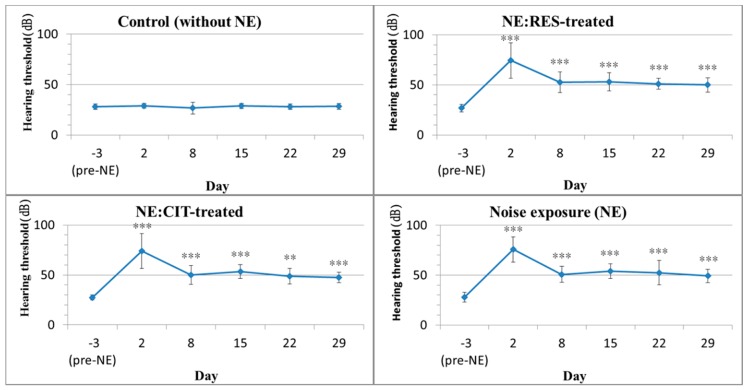
The four graphs depict the effects of noise exposure on the hearing threshold. Mean ABR thresholds in response to click stimuli are displayed for the control group (n = 6; upper left), the RES-treated group (n = 6; upper right), the CIT-treated group (n = 6; lower left), and the NE group (n = 6; lower right) before and after exposure to narrowband noise (8 kHz, 116 dB) for 3.5 h. After noise exposure, a permanent hearing loss of approximately 50 dB SPL was seen in the NE and the drug-treated groups during the following 4 weeks. * *p* < 0.05, ** *p* < 0.01 and *** *p* < 0.001 versus the control group. (ABR: auditory brainstem response, CIT: citalopram, RES: resveratrol, NE: noise exposure).

Before noise exposure, the hearing thresholds of rats in all groups were 27.5 ± 3.6 dB. After exposure to narrowband noise (8 kHz, 116 dB) for 3.5 h, the hearing thresholds of rats in all noise exposure (NE) groups with or without drug treatment were elevated to 70.6 ± 15.8 dB on day 2 and recovered to approximately 51.3 ± 7.9 dB on day 8 until day 29, when the last auditory brainstem response (ABR) test was performed (Figure 1). The hearing threshold of the control group was stable at approximately 25 dB throughout the experimental process. However, based on the actin staining of outer hair cells on the organ of Corti, no substantial loss of outer hair cells was noted in any of the four groups at 4 weeks (Supplementary Figure S1), suggesting that this noise-induced impairment in hearing capability may not have resulted from physical damage to the hair cells.

### 2.2. Noise Exposure Decreased SERT Levels in Multiple Brain Regions

**Figure 2:**
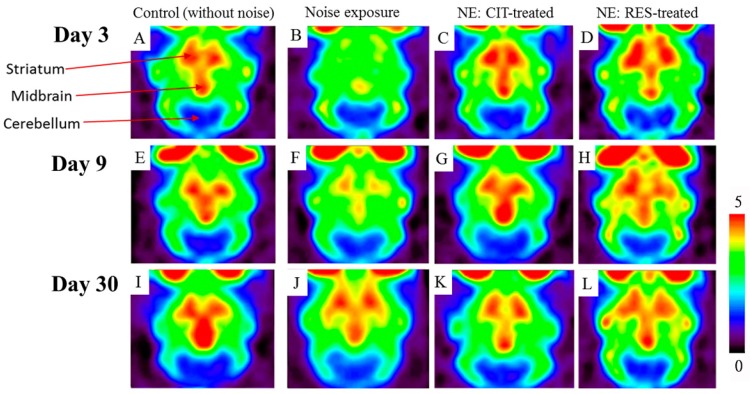
4-[^18^F]-ADAM/small-animal PET images of transverse sections are displayed. Images were acquired from the control group (**A**,**E**,**I**), NE group (**B**,**F**,**J**), CIT-treated group (**C**,**G**,**K**), and RES-treated group (**D**,**H**,**L**) on day 3 (**A**–**D**), day 9 (**E**–**H**), and day 30 (**I**–**L**). The PET images from the NE group clearly portray less 4-[^18^F]-ADAM uptake relative to the other groups on days 3 and 9. The color scale for all images was adjusted by the standardized uptake value of cerebellum as reference region. The standardized uptake value ratio is the unit of the color scale (0~5). (CIT: citalopram, RES: resveratrol, NE: noise exposure, PET: positron emission tomography).

**Figure 3:**
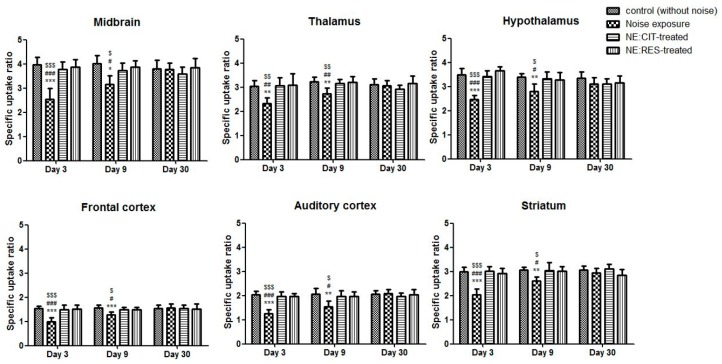
The graphs portray the SURs of 4-[^18^F]-ADAM/micro-PET on days 3, 9, and 30 in six brain regions. The SURs of 4-[^18^F]-ADAM were significantly lower in the NE group compared with the control group and the CIT-/RES-treated groups on days 3 and 9. There were no significant differences among the groups on day 30. * *p* < 0.05, ** *p* < 0.01, *** *p* < 0.001 for comparisons between the control group and NE group; ^$^ *p* < 0.05, ^$$^ *p* < 0.01, ^$$$^ *p* < 0.001 for comparisons between the NE group and CIT-treated group; ^#^ *p* < 0.05, ^##^ *p* < 0.01, ^###^ *p* < 0.001 for comparisons between the RES-treated group and NE group. (SUR: specific uptake ratio, CIT: citalopram, RES: resveratrol, NE: noise exposure).

4-[^18^F]-ADAM/PET imaging, for assessing availability of SERT, was performed after noise exposure on days 3, 9, and 30. Compared with the control group, the SERT availabilities in various brain regions were decreased in the NE group (Figure 2). In the NE group, on day 3, the average specific uptake ratio (SUR) values for SERT quantitation in tested brain regions were reduced by 23–39% compared with those in the control group, and these remained at a 15–24% decrement on day 9 (Figure 3). Four weeks after noise exposure, the PET images and SUR values of the NE group revealed recovery of 4-[^18^F]-ADAM uptake when compared with uptake in the control group (Figure 2 and Figure 3).

**Figure 4:**
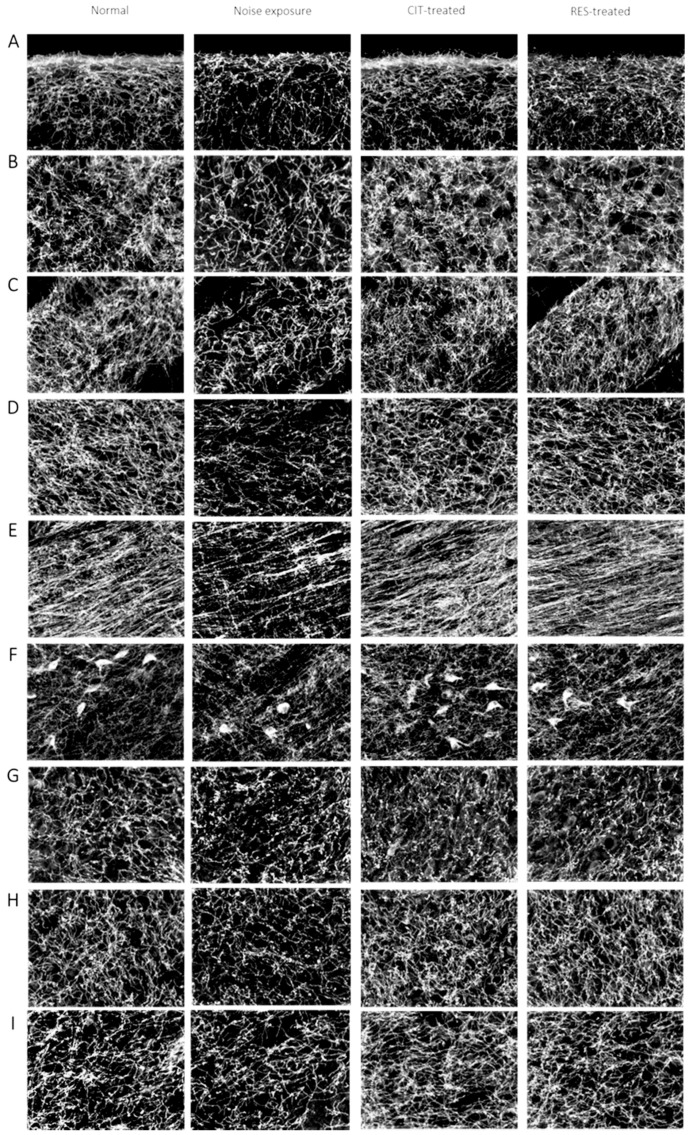
Photomicrographs of serotonin transporters following immunohistochemistry staining in various brain regions of the control group, the NE group, the CIT-treated group, and the RES-treated group 4 days after noise exposure are displayed. The serotonergic fiber densities in various brain regions were lower in the NE group. (**A**) frontal cortex; (**B**) auditory cortex; (**C**) striatum; (**D**) thalamus; (**E**) hypothalamus; (**F**) raphe nucleus; (**G**) cochlear nucleus; (**H**) inferior colliculus; and (**I**) hippocampus. (CIT: citalopram, RES: resveratrol, NE: noise exposure).

**Figure 5:**
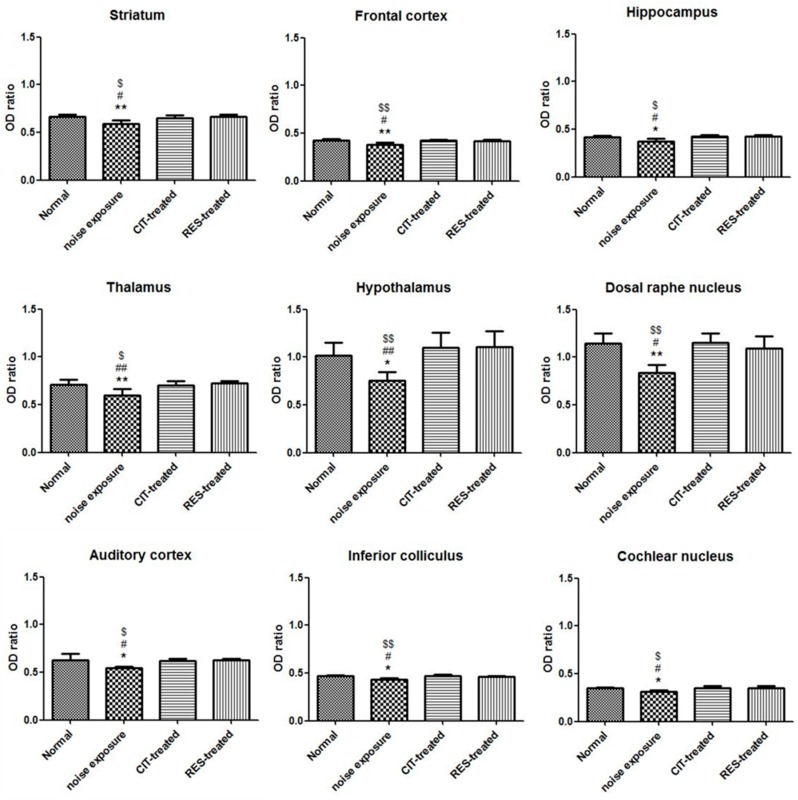
The graphs display OD ratios in various brain regions at day 4 after noise exposure. The OD ratios in various brain regions were significantly lower than those of the NE group. * *p* < 0.05, ** *p* < 0.01, *** *p* < 0.001 for comparisons between the control group and NE group; ^$^ *p* < 0.05, ^$$^ *p* < 0.01, ^$$$^ *p* < 0.001 for comparisons between the NE group and CIT-treated group; ^#^ *p* < 0.05, ^##^ *p* < 0.01, ^###^ *p* < 0.001 for comparisons between the RES-treated group and NE group. (OD: optical density, CIT: citalopram, RES: resveratrol, NE: noise exposure).

We also conducted immunohistochemistry (IHC) for SERT on days 4 and 31 after noise exposure. High densities of SERT-immunoreactivity (SERT-ir) fibers were present in various brain regions in the control group. In the NE group, a mild decrease (7–15%) in SERT-ir fibers was observed in the brain regions of the auditory pathway, including the cochlear nucleus, inferior colliculus, thalamus, and auditory cortex, compared with the control group on day 4. In addition, in nonauditory brain regions of the NE group, including the striatum, hippocampus, hypothalamus, and dorsal raphe nucleus, optical density (OD) ratios of SERT-ir were also reduced by 10–27% compared with the control group (Figure 4 and Figure 5). However, no differences in the OD ratios of SERT-ir between the control and NE groups on day 31 were noted (Supplementary Figures S2 and S3).

### 2.3. Resveratrol Conferred Neuroprotection against Noise-Induced SERT Loss

The results of 4-[^18^F]-ADAM/PET imaging showed that both citalopram and resveratrol prevented noise-induced SERT damage after noise exposure on days 3 and 9 compared with the NE group (Figure 2 and Figure 3).

IHC of the drug-treated groups was performed after noise exposure on days 4 and 31. Analysis of the IHC images and OD ratios revealed that citalopram and resveratrol restored noise-induced SERT-ir fiber damage compared with the NE group on day 4 (Figure 4 and Figure 5), whereas no differences were found between the four groups on day 31 (Supplementary Figures S2 and S3).

## 3. Discussion

Cochlear serotonergic innervation is constituted by efferent fibers projecting to the inner and the outer hair cells. Previous studies showed that the existence of serotonergic synaptic activity in the cochlea by measuring the levels of 5-HT metabolites and 5-HT receptor mRNAs [25,26]. 5-HT is also suggested to play a modulatory role in hearing and balance by modulating the afferent fiber discharge rate in the inferior colliculus, auditory cortex, and other nuclei of the ascending auditory system [27,28,29,30,31]. Because resveratrol and citalopram may block the SERT-mediated reuptake of 5-HT and consequently increase the synaptic levels of 5-HT, we hypothesized that the increment of synaptic 5-HT levels would reduce the noise-induced excitotoxicity of afferent auditory nerves by modulating glutamate neurotransmission through the 5-HT receptors in the brain regions of the auditory pathway. We found that resveratrol and citalopram may accelerate SERT recovery from noise-induced damage in the rat brain in vivo despite hearing not being rescued by both drugs. 

There are two types of afferent neurons (spiral ganglion cells). The majority (95%) are type I afferents which contact inner hair cells and sensitive to sound [32,33]. The tyrosine hydroxylase-expressing type II afferents (5%), functioning as cochlear nociceptors, receive input from outer hair cells but are insensitive to sound [34,35]. In the cochlea, noise-induced glutamate excitotoxicity is characterized by a two-step mechanism. First, acoustic overexposure causes the acute swelling of type I afferent neuronal terminals in the region of their synaptic contact with inner hair cells, resulting in a temporary threshold shift. Within the next few days, synaptic repair is observed with a full or a partial recovery of cochlear potentials [36,37]. These observations are consistent with the ABR data in this study. Hearing thresholds were temporally elevated and partially recovered later after noise exposure regardless of treatment with citalopram or resveratrol, which suggests that these drugs could not attenuate noise-induced hearing loss in the first phase of noise-induced glutamate excitotoxicity. In the second phase, glutamate excitotoxicity further causes spiral ganglion neuronal death primarily through the excessive activation of glutamate receptors, which triggers a massive Ca^2+^ influx into neurons [38], resulting in the production of reactive oxygen species (ROS) and free radicals by mitochondria and, eventually, cell death [39]. Therefore, such changes of spiral ganglion cells are likely to alter the transmission of information in the ascending auditory pathway, and presumably contribute to the serotonergic neurotoxicity in the central nervous system. The results of PET imaging with 4-[^18^F]-ADAM in this study revealed that exposure to excessive noise rapidly reduced the SERT availability in most brain regions of the rats in the NE group, and the reductions persisted at least 1 week after noise exposure, which is in agreement with our IHC data. Given that SERT is a presynaptic integral membrane protein expressed in the serotonergic system and a critical indicator of the integrity of serotonergic neurons [40], we postulated that noise-induced glutamate excitotoxicity may also reduce innervation of serotonergic neurons. 

As both resveratrol and citalopram could bind SERT to increase synaptic 5-HT levels [21,41], both drugs may inhibit noise-induced excitatory glutamate transmission and ameliorate consequent cell death of serotonergic neurons. In this study, resveratrol and citalopram ameliorated SERT reduction on days 3 and 9 after noise exposure. These results suggest that these SERT inhibitors provide protection against noise-induced SERT loss, as detected using in vivo 4-[^18^F]-ADAM/small-animal PET and IHC. In addition, resveratrol decreased intracellular ROS production, which protected neurons from glutamate-induced neuronal damage, likely by mechanisms involving the NMDA receptor-intracellular Ca^2+^ pathway [42]. Moreover, due to the redox properties of its phenolic hydroxy groups and the potential for electron delocalization across the chemical structure, resveratrol can scavenge both HO• and O_2_•^−^ radicals to reduce oxidative stress in the mitochondria of neurons [43,44]. Therefore, resveratrol can provide protection against noise-induced SERT loss by not only blocking synaptic 5-HT uptake but also by regulating the production of ROS and scavenging free radicals. 

Our IHC data revealed that the OD ratios of the global brain SERT-ir fibers decreased after day 4 in the NE group compared with the control group, but the resveratrol or citalopram-treated rats had similar fiber density of SERT-ir compared with the control group. After a total of 4 weeks noise exposure, no differences were observed in the OD ratios among the four groups. Kang et al. used a 118 dB SPL and 8-kHz narrowband noise for 3.5 h in a soundproof chamber and found that whole brain SERT availability recovered after 1 month, but not to entirely normal levels. The remaining decrements were 26.1% in the midbrain and 47.7% in the frontal cortex [5]. In our experimental design, we replaced the 118 dB SPL with 116 dB, for a noise intensity that was 60% of the previous study, which may explain the disparate results. The 116-dB noise level caused serotonergic nerve fiber trauma and a decrease in brain SERT; however, serotonergic fibers were still recovered in the tested brain regions after 1 month (Supplementary Figure S2). One possible explanation is that when a loud noise causes inner hair cell or afferent nerve fiber impairment, the neighboring intact fibers extend to the impaired region to cause reorganization or growth [45]. It has also been reported that SERT located on the presynaptic neuron membrane could cause internalization by phosphorylation when the nerve is stimulated [46]. Stimulation by a strong noise may result in SERT internalization and dephosporylation to force the membrane to rid itself of the noisy environment, which may explain why the images and OD values of the brain SERT gradually recovered after noise exposure.

The serotonin system has an association with tinnitus. Serotonin, an inhibitory neurotransmitter, has been proposed to play an intermediary role in expressing plasticity following acoustic trauma and to regulate the balance between excitation and inhibition of central auditory circuits [47,48]. In addition, the long/long genotype variant of the SERT promoter region, which increases 5-HT reuptake in synapses and leads to 5-HT depletion, seems to be associated with the limbic and autonomic nervous system symptoms of patients with tinnitus [49]. These findings imply that serotonin replacement or serotonin reuptake inhibitors may increase the success rate of tinnitus treatment modalities. If serotonin availability corresponds to serotonergic fiber density, our IHC data revealed that acoustic trauma could induce loss of serotonergic fibers, which may consequently decrease synthesis of 5-HT to result in lower synaptic 5-HT levels. Therefore, we propose that both resveratrol and citalopram may help prevent noise-induced tinnitus through modulation of synaptic 5-HT levels.

In conclusion, we found that narrowband noise at 116 dB and 8 kHz for 3.5 h in a soundproof chamber not only elevated the hearing threshold of rats but also affected their expression of SERT. Our results suggest that resveratrol and citalopram have protective effects against noise-induced loss of SERT in spite of neutral effects on the hearing threshold.

## 4. Materials and Methods

### 4.1. Experimental Animals

All animal experiments were approved by the Institutional Animal Care and Use Committee of the National Defense Medical Center (Taipei, Taiwan). The procedures for animal care complied with institutional guidelines and regulations (approval no. IACUC-14-045). Eight-week-old male Sprague-Dawley rats (250–300 g in weight) were housed in the animal center at the National Defense Medical Center and kept at a constant temperature of 23 ± 2 °C with controlled light–dark cycles (light from 7:00 AM to 7:00 PM). 

The rats were divided into four groups (n = 6 per group): a control group (saline-treated, intraperitoneal injection [IP], without noise exposure), a noise exposure group (NE; saline-treated, IP), a noise exposure with citalopram treatment group (NE:CIT-treated; 20 mg/kg citalopram, IP), and a noise exposure with resveratrol treatment group (NE:RES-treated; 30 mg/kg resveratrol, IP). In a preliminary experiment (data not shown), we tested rats with different intensity of noise at 114 dB, 116 dB and 118 dB SPL. We found that in rats with 114 dB SPL noise, there was mild elevation of hearing threshold from 20 dB to 40 dB in ABR tests. However, 118 dB SPL noise induced irreversible elevation of hearing threshold to 90 dB (reach the cutoff for the highest intensity measured during the assay). Therefore, in this study, we used the moderate intensity of noise at 116 dB to examine the effects of drugs. All groups except for the control group were exposed to noise (narrowband noise [8 kHz, 116 dB]) for 3.5 h under general anesthesia in a soundproof chamber on day 0. The rats were treated with saline, citalopram, or resveratrol once a day for four successive days from days −2 to 2. Citalopram (Toronto Research Chemicals Inc., North York, ON, Canada) was dissolved in saline (0.9% NaCl) at a concentration of 20 mg/kg and administered through IP. Resveratrol (Sigma-Aldrich, St. Louis, MO, USA) was dissolved in 20% alcohol and administered through IP at a concentration of 30 mg/kg.

**Figure 6:**
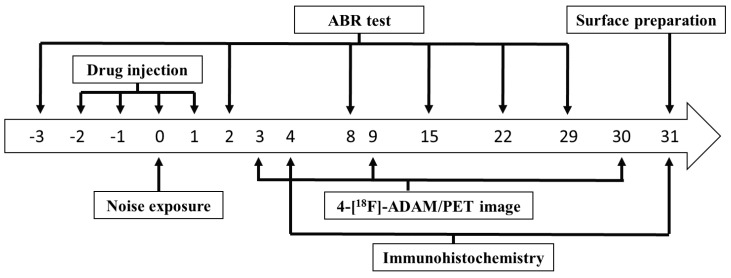
The diagram depicts the experimental schedule. Rats were exposed to noise on day 0. ABR tests were performed on days −3, 2, 8, 15, 22, and 29. 4-[^18^F]-ADAM/PET images were performed on days 3, 9, and 30. Immunohistochemistry was performed on days 4 and 31. Surface preparation was performed on day 31. (ABR: auditory brainstem response).

Hearing thresholds were assessed on days −3, 2, 8, 15, 22, and 29. Small-animal PET with 4-[^18^F]-ADAM was performed on days 3, 9, and 30. Surface preparation of outer hair cells in the cochlea was conducted on day 31. IHC for various brain areas was performed on days 4 (different rats receiving the same protocol) and 31. The schedule of the experiments is presented in Figure 6.

### 4.2. Hearing Threshold Detection

Hearing thresholds were assessed in all groups using click stimuli as the ABR test, as described previously [50]. Briefly, rats were anaesthetized and subdermal needle electrodes were inserted at the vertex (negative), underneath the pinna of the ear (positive), and on the back (ground) in a soundproof chamber. We analyzed the sound intensity and recorded the hearing thresholds until the data showed no response for the sound stimuli. The lowest value was recorded and defined as the hearing threshold.

### 4.3. Cochlear Surface Preparation and Actin-Staining

Rat tympanic bullae were dissected and fixed overnight in 4% paraformaldehyde at 4 °C. Tissues were immersed in 10% ethylenediaminetetraacetic acid for one month. The tympanic bulla, lateral wall, Reissner’s membrane, and tectorial membrane of the cochlea were removed. The organ of Corti was left in place and stained with phalloidin (1:200; Invitrogen, Carlsbad, CA, USA) for 1 h. According to the various locations (3.3 mm from apex) that corresponded to 8 kHz [51], the organ of Corti was dissected into three fragments, mounted on gelatin-coated glass slides, and examined with a Zeiss LSM 510 confocal microscope (Carl Zeiss, Jena, Germany) equipped with a 40× objective lens. Images were analyzed with Meta Morph image analysis software (MDS Analytical Technologies, Downingtown, PA, USA) to evaluate hair cell survival.

### 4.4. Small Animal-PET Imaging

4-[^18^F]-ADAM (purity > 95%; specific activity > 3 Ci/μmol) was synthesized as previously reported by the Department of Nuclear Medicine at the Tri-Service General Hospital, Taiwan. Small-animal PET image acquisition was performed as previously described [52,53]. First, rats were injected with 4-[^18^F]-ADAM (14.8–18.5 MBq) through the tail vein. The acquisition time of PET images was 60–90 min after radioligand injection. Data of PET images were reconstructed using a micro-PET R4 scanner (Concorde MicroSystems, Knoxville, TN, USA). 

The quantitative method for micro-PET imaging with 4-[^18^F]-ADAM has been reported in our previous studies [24,52,53]. In brief, to minimize inconsistencies in volume of interest (VOI) placement among the animals, the MR images were obtained from a typical SD rat brain and fused manually with 6 reconstructed 4-[^18^F]-ADAM PET images of normal SD rats to draw VOIs according to a rat brain atlas [54]. The typical MR images with the VOIs were saved as a template for further analysis. The 4-[^18^F]-ADAM images of each individual animal in this study were co-registered manually to the corresponding MR template images using ASIPro VM 6.3.3.1 software (Concorde MicroSystems, Knoxville, TN, USA) for measuring standardized uptake value (SUV) in various brain regions. The final data were expressed as specific uptake ratios (SURs) [53], calculated as SUR = (SUV_target region_ − SUV_cerebellum_)/SUV_cerebellum_.

### 4.5. Immunohistochemistry

IHC was performed as previously described [55,56,57,58] with minor modifications. Rat brains were fixed in 4% paraformaldehyde in phosphate-buffered saline (PBS, 0.1 M), cryoprotected overnight in a solution of 20% sucrose in PBS (0.1 M) at 4 °C, and then changed to a 30% sucrose solution at 4 °C overnight. The brains were sliced using a cryostat microtome (Leica CM 3050; Leica Microsystems, Wetzlar, Germany), and sagittal sections (30 μm) were rinsed in PBS and then incubated in 1% H_2_O_2_ in PBS for 40 min. After being washed three times, the brain sections were incubated in blocking solution (1% normal goat serum in 0.1 M PBS with 0.5% Triton X-100) for 1 h. Then, rabbit anti-SERT antibody (1:2000; Millipore Corporation, Middlesex, MA, USA) was applied over two nights at 4 °C. The brain sections were further incubated with goat antirabbit biotinylated IgG (1:200; Vector, Torrance, CA, USA) and avidin-biotin complex (1:200; Vectastain ABC kit, Vector, Kentland, IN, USA). Finally, the brain sections were washed, dried, and mounted on gelatin-coated glass slides.

Optical density (OD) was defined as the average of the ODs measured in three target brain regions taken from consecutive brain sections. All images of target regions and reference region (corpus callosum) were converted to eight-bit grey scale (0–255 grey levels) and analyzed using Image-Pro Plus v6.0 analysis software (Media Cybernetics, Inc., Rockville, MD, USA) to obtain SERT immunoreactivity (SERT-ir) OD values [59,60]. The OD ratio of the target region relative to the reference region was calculated using the formula: OD ratio = (OD_target region_ − OD_corpus callosum_)/OD_corpus callosum_.

### 4.6. Statistical Analysis

Results are expressed as mean ± standard deviation. Data were analyzed using one-way analysis of variance (ANOVA) or two-way repeated measures ANOVA, followed by a post hoc Bonferroni test of honestly significant difference by GraphPad Prism 8.0 (GraphPad Software Inc, San Diego, CA, USA). The α level for a type I error was set at 0.05 for two-tailed tests of significance.

## Supplementary Materials

The following are available online at, Figure S1: The photos depict the effect of noise exposure and drug protection on outer hair cell survival. No substantial losses of outer hair cells were noted following noise exposure for 4 weeks. (**A**) control group; (**B**) noise exposure group; (**C**) resveratrol-treated group; (**D**) citalopram-treated group., Figure S2: Photomicrographs of serotonin transporters by immunohistochemistry stain in various brain regions of the control group, noise exposure group, citalopram-treated group, and resveratrol-treated group are displayed. The serotonergic fiber densities in various brain regions were not different among the groups. (**A**) frontal cortex; (**B**) auditory cortex; (**C**) striatum; (**D**) thalamus; (**E**) hypothalamus; (**F**) raphe nucleus; (**G**) cochlear nucleus; (**H**) inferior colliculus; and (**I**) hippocampus, Figure S3: The graphs display optical density (OD) ratios in various brain regions at 4 weeks after noise exposure. The OD ratios in various brain regions were not different among the control group, noise exposure group, and drug-treated groups at 4 weeks after noise exposure.
