# Supplementary material for: Regulation of Noise-Induced Loss of Serotonin Transporters with Resveratrol in a Rat Model Using 4-[18F]-ADAM/Small-Animal Positron Emission Tomography
Source: Molecules. 2019 Apr 5;24(7):1344. doi: 10.3390/molecules24071344 (PMC6480549; doi:10.3390/molecules24071344)
Supplement: Supplementary file 1 [file molecules-24-01344-s001.pdf]

Regulation of Noise-Induced Loss of Serotonin Transporters with Resveratrol in a Rat Model Using 4-[^{18}F]-ADAM/Small-Animal Positron Emission Tomography

I-Hsun Li [1,2], Jui-Hu Shih [1,2], Yun-Tin Jhao [3], Hsin-Chien Chen [4], Chuang-Hsin Chiu [5], Chien-Fu F. Chen [6], Yuahn-Sieh Huang [3], Chyng-Yann Shiue [5] and Kuo-Hsing Ma [3,*]

1 Department of Pharmacy Practice, Tri-Service General Hospital, Taipei, Taiwan; lhs01077@gmail.com (I.-H.L.); jtlovehl@gmail.com (J.-H.S.)
2 School of Pharmacy, National Defense Medical Center, Taipei, Taiwan
3 Department of Biology and Anatomy, National Defense Medical Center, Taipei, Taiwan; ytcola581470@gmail.com (Y.-T.J.); anatoman2001@yahoo.com.tw (Y.-S.H.)
4 Department of Otorhinolaryngology-Head and Neck Surgery, Tri-Service General Hospital, National Defense Medical Center, Taipei, Taiwan; acolufreia@yahoo.com.tw
5 Department of Nuclear Medicine, Tri-Service General Hospital, National Defense Medical Center, Taipei, Taiwan; treasure316@gmail.com (Y.-S.H.); shiue@ntuh.gov.tw (C.-Y.S.)
6 Graduate Institute of Life Sciences, National Defense Medical Center, Taipei, Taiwan; t70cyy@yahoo.com
* Correspondence: kuohsing91@yahoo.com.tw; Fax: +886-2-87923159

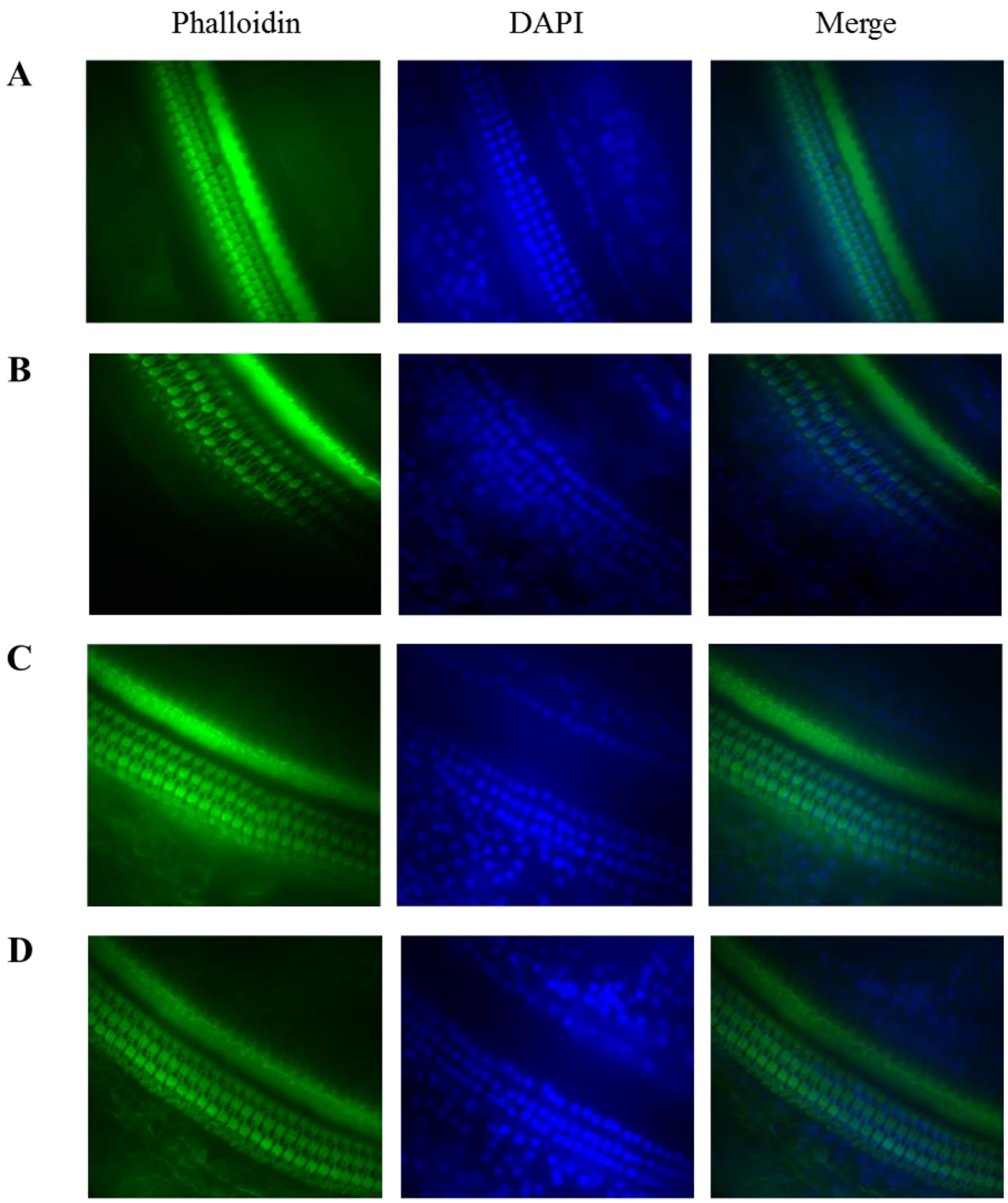

Supplementary Figure S1. The photos depict the effect of noise exposure and drug protection on outer hair cell survival. No substantial losses of outer hair cells were noted following noise exposure for 4 weeks. (**A**) control group; (**B**) noise exposure group; (**C**) resveratrol-treated group; (**D**) citalopram-treated group.

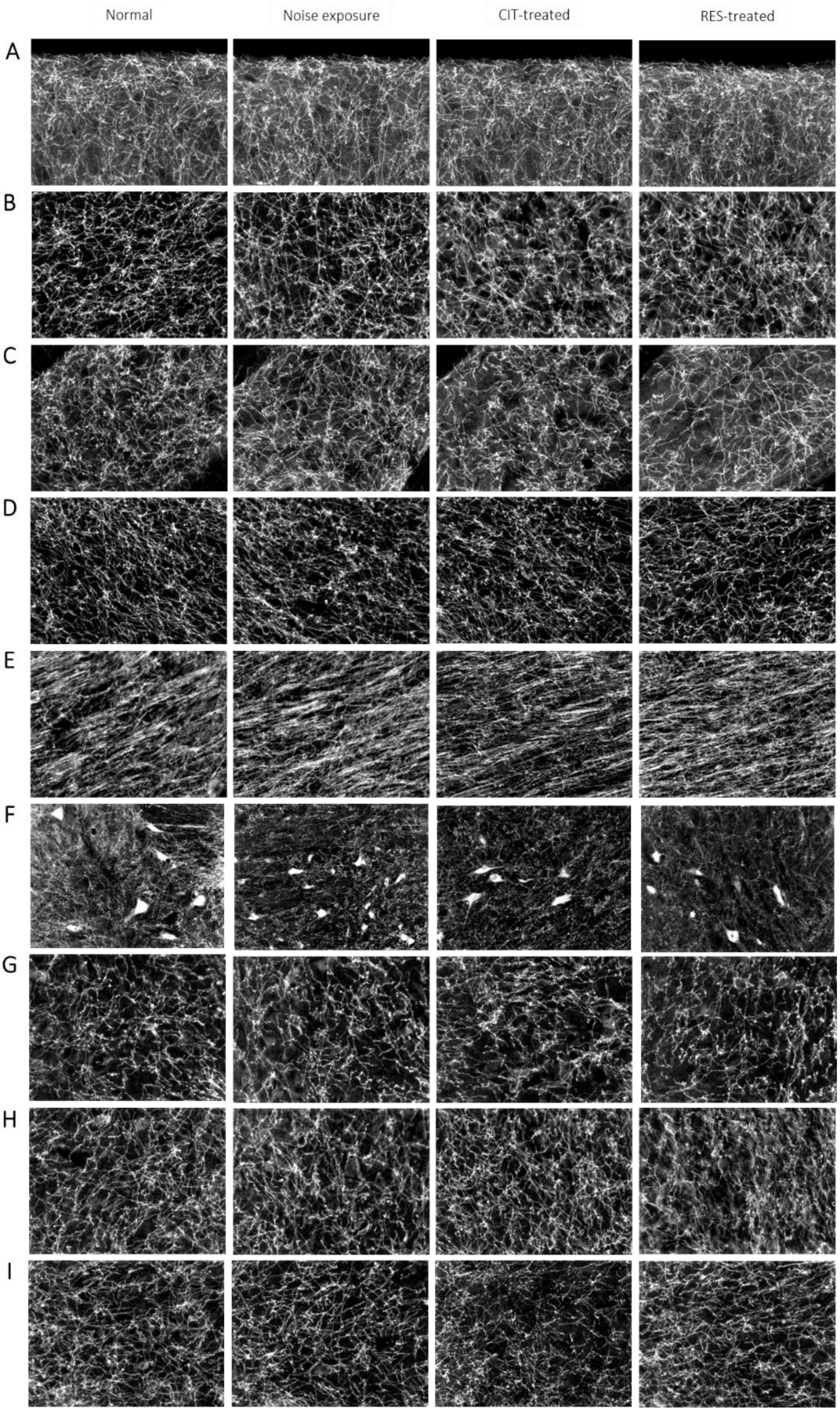
Normal
Noise exposure
CIT-treated
RES-treated
A
B
C
D
E
F
G
H
I

Supplementary Figure S2. Photomicrographs of serotonin transporters by immunohistochemistry stain in various brain regions of the control group, noise exposure group, citalopram-treated group, and resveratrol-treated group are displayed. The serotonergic fibre densities in various brain regions were not different among the groups. (**A**) frontal cortex; (**B**) auditory cortex; (**C**) striatum; (**D**) thalamus; (**E**) hypothalamus; (**F**) raphe nucleus; (**G**) cochlear nucleus; (**H**) inferior colliculus; and (**I**) hippocampus**.**

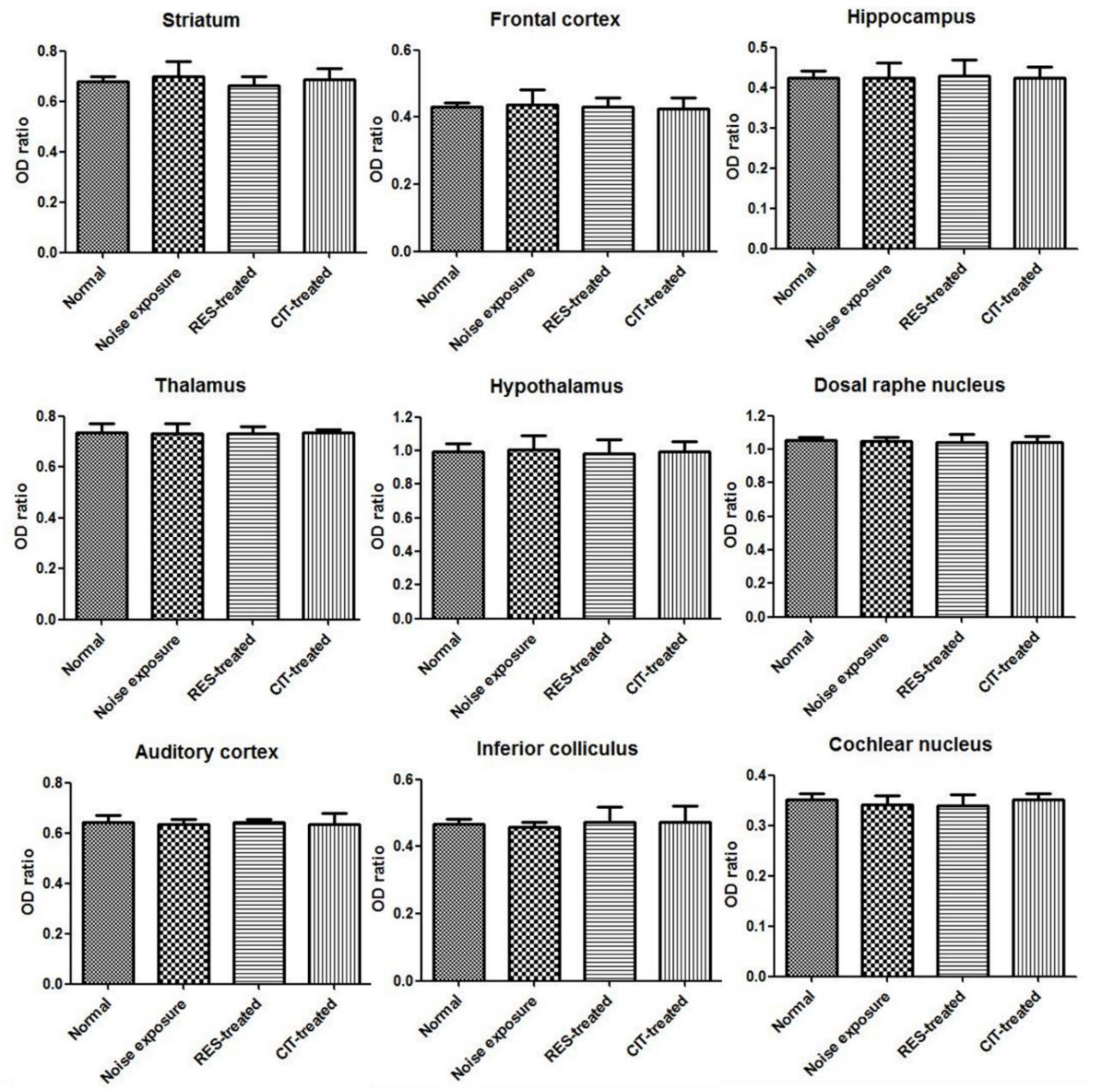

Supplementary Figure S3. The graphs display optical density (OD) ratios in various brain regions at 4 weeks after noise exposure. The OD ratios in various brain regions were not different among the control group, noise exposure group, and drug-treated groups at 4 weeks after noise exposure.
